# Evaluating the transition of adolescents and young adults with palliative care needs from pediatric to adult care

**DOI:** 10.1016/j.hctj.2024.100072

**Published:** 2024-09-14

**Authors:** Emma W. Healy, Natasha Z. Piracha

**Affiliations:** a Columbia University Vagelos College of Physicians and Surgeons, 630 W 168th St, New York, NY 10032, United States; b Pediatric Palliative Care, Division of Critical Care and Hospital Medicine, Department of Pediatrics, Columbia University Vagelos College of Physicians and Surgeons and New York-Presbyterian, 630 W 168th St, New York, NY 10032, United States; c Adult Palliative Care Service, Department of Medicine, Columbia University Vagelos College of Physicians and Surgeons and New York-Presbyterian, 630 W 168th St, New York, NY 10032, United States

**Keywords:** Healthcare transitions, Palliative care, Chronic disease, Young adult, Goals of care

## Abstract

**Background:**

The transition from pediatric to adult healthcare poses significant challenges for adolescents and young adults (AYA), especially those with chronic conditions, yet most children receive inadequate transition preparation. Research on the transition for patients receiving palliative care services is particularly limited. We sought to address this gap in the literature.

**Methods:**

Young adults aged 18 to 35 years who transitioned from the pediatric setting and received adult palliative care services at an urban academic medical center between the dates of February 1st_,_ 2020 and July 1st, 2022 were identified retrospectively via electronic medical record. Chart review was used to investigate outcomes of interest, including use of pediatric palliative care services and timing of care conversations.

**Results:**

Only 23 % of patients interfaced with pediatric palliative care, despite all having childhood diagnoses. Pediatric palliative care exposure was associated with a significantly earlier median age of first adult palliative care encounter (19.63 versus 25.06, p = <0.001). Goals of care discussions, code status conversations, and healthcare proxy documentation occurred earlier if pediatric palliative care was involved (18.9 years versus 25.7 years, p < 0.001; 20.9 years versus 30.0 years, p < 0.001; 20.7 versus 28.9, p < 0.001).

**Conclusions:**

Pediatric palliative care services were underutilized in AYA patients, but when used, were associated with earlier adult palliative care encounters, goals of care discussions, code status decisions, and health care proxy identification.

## Highlights

• Transitioning to adult care is difficult for young patients with complex illness.
• Research on the role of palliative care in the transition to adulthood is limited.
• Palliative care services are underutilized in adolescent and young adult patients.
• Pediatric palliative care services facilitate earlier goals of care discussions.
• Pediatric palliative care involvement expedites connection to adult services.

## Graphical Abstract

**Figure:**
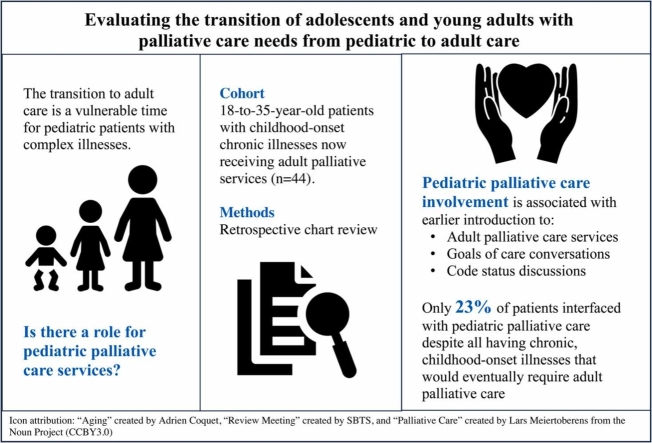

## 1 Introduction

The transition from pediatric to adult healthcare poses significant challenges for adolescents and young adults (AYA), especially those with complex and chronic conditions, and yet most children receive inadequate transition preparation.^1^, ^2^, ^3^ Insufficient support during the transition process is associated with several adverse effects, including discontinuity of care, higher rates of medical complications, patient dissatisfaction, lower treatment adherence, increased acute service utilization, and higher overall costs of care.^4^ In contrast, implementing a structured approach to the transition process has positive effects, such as improved adherence to care, visit attendance, and patient-reported quality of life, as well as decreased rates of hospitalization and shorter gaps between pediatric and adult visits.^5^

Research on the transition of care for patients receiving palliative care services is limited. Current research has demonstrated unmet needs: one systemic review of studies on AYA patients who had conditions appropriate for palliative care and who were transitioning from pediatric to adult services found palliative care to be an understudied resource.^6^ This analysis found only three papers, all non-empirical, that used the term “palliative care” in relation to the transition to adult services.^6^ The authors also found a general lack of structured transition programs and a dearth of long-term outcome data to compare the effectiveness of models that did exist.^6^ One qualitative study centered around interviews with AYA hospice patients found that transition planning was absent or poorly coordinated.^7^ The topic of how to adequately transition young patients with chronic or life-limiting illnesses into adulthood is especially important as more individuals with illnesses that were previously fatal in childhood survive into adulthood.^8^

To address this gap in the literature, we identified young adult patients receiving palliative care services who had transitioned from the pediatric setting and retrospectively analyzed their transitions. Our primary goal was to investigate pediatric palliative care utilization, exploring how it affected continuity of care for adolescents with conditions appropriate for palliative care as they transitioned from childhood to adulthood. For the purposes of this study, any condition for which a patient was accepted to be seen at our palliative care clinic (as determined by care manager review) was considered a condition appropriate for palliative care. These included multiple severe medical conditions including hematological disorders, cancers, neurological disorders, genetic disorders, and other conditions.

## 2 Materials and methods

### 2.1 Study design and population

**Fig. 1:**
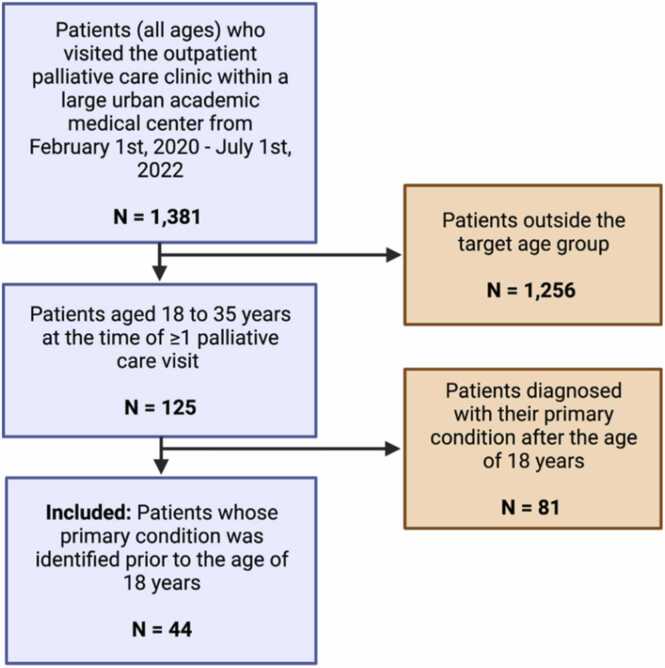
Inclusion criteria.

Using the electronic medical record (EMR), we queried a list of all patients who visited our institution’s adult palliative care clinic between the dates of February 1st_,_ 2020 and July 1st, 2022, generating a list of 1381 patients. We narrowed the list to young adult patients aged 18 to 35 (125 patients) (Fig. 1). We did not focus on a specific medical condition—such as sickle cell disease, cancer or cystic fibrosis—as several institutions have condition-specific programs and resources, and our primary outcome of interest was utilization of pediatric palliative care services more broadly. Additionally, the study population was not narrowed based on life-expectancy or reason for referral to palliative care, as our goal was to provide broad and descriptive information, given the current lack of research on this topic.

To isolate patients who had transitioned from pediatric to adult services, only individuals whose primary condition was identified prior to the age of 18 were included in our cohort. Patients who were diagnosed after the age of 18—the age at which our adult palliative care clinic starts accepting patients—were excluded (81 patients) (Fig. 1). The final study population included 44 patients.

We used retrospective chart review to describe the population and investigate the success of their transitions. Our primary outcome of interest was involvement of pediatric palliative care. Our secondary outcomes were chosen based on the most frequently used palliative care and transition care outcome metrics cited in the literature.^9^, ^10^, ^11^, ^12^ We recorded the percent of patients who had documented discussions about their goals of care (GOC), code status, and advanced directives—as well as the ages at which these first conversations occurred. We assessed in-hospital mortality, the pathways through which patients were referred to palliative care, and the transition from pediatric to adult palliative services. Additionally, we investigated rates of missed appointments, acute care service utilization, and loss to follow up.

### 2.2 Data collection

Data were collected via retrospective chart review through the EMR. Only documentation that was available to view within our hospital network (NewYork-Presbyterian) was included in the analysis. Records from October 31st, 1997, to October 31st, 2022, were included in the analysis.

### 2.3 Statistics

Clinical and demographic variables were reported using standard summary statistics: continuous variables were expressed as medians (with interquartile range) or means (with standard deviation), depending on normality, and categorical variables were expressed as percentages. Normality was evaluated via Shapiro-Wilk test. Parametric variables were compared using t-tests.

Unadjusted event rates were calculated by dividing the number of events (e.g., emergency department (ED) visits, hospitalizations, inpatient days, total acute care encounters, and missed appointments) by the amount of time (in years) that each patient was followed. The boundaries of this period started when a participant had their first visit documented in the EMR and ended when a participant died, or the data collection period ceased. If a patient was directly admitted from the ED, this encounter was documented only as a hospital admission to avoid duplicate entries*.* Event rates were calculated for the periods before and after each participant turned 21 years old (set using the birth dates documented in the EMR), and these rates were compared using paired-sample sign tests. We chose the age of 21 years for pre- versus post-transition analyses, as it is the age at which our institution’s pediatric ED and inpatient hospital stop accepting patients.

Kaplan-Meier curves were used to estimate the time to an event (reported in years of age) for certain outcomes of interest not completed by all participants during the study period (e.g., GOC discussion, code status conversation). Comparisons of survival curves were done via log rank test. Some variables (e.g., mortality, documentation of advance directives) had insufficient events to perform a time-to-event analysis and were described as medians or averages, as described above. All analyses were performed using R Statistical Software (version 3.4.3; Vienna, Austria).

### 2.4 IRB approval

Approval was granted by the Institutional Review Board of Columbia University Medical Center (7/16/2022, Protocol #AAAU2001).

## 3 Results

### 3.1 Demographics

Forty-four patients were included in our analysis. Of these patients, 52 % (N = 23) identified as male, 46 % (N = 20) as female, and 2 % (N = 1) as nonbinary (Table 1). Forty-eight percent (N = 21) of patients self-identified as White, 34 % (N = 15) as African American or Black, 7 % (N = 3) as Asian, and 2 % (N = 1) as American Indian or Alaskan Native. Forty-eight percent (N = 21) of patients self-identified as Hispanic or Latino (Table 1). Seventy-three percent of patients identified with a religion (59 % Christian, 7 % Jewish, 5 % Islam, and 3 % Hindu) (Table 1). Fifty-nine percent of patients were diagnosed with their primary condition prenatally or at birth. The median age of diagnosis for all patients (N = 44)—including those diagnosed perinatally—was 0 (IQR: 0–14) years. For patients who were diagnosed after birth (N = 18), the median age of diagnosis was 16 (IQR: 7.25–18) years. The most common primary diagnoses in our sample of patients were sickle cell disease (32 %, N = 14)), oncological diseases (20 %, N = 9), birth defects (16 %, N = 7), genetic syndromes (11 %, N = 5), cystic fibrosis (7 %, N = 3), and chronic pancreatitis (7 %, N = 3) (Table 1).

**Table 1:** Patient demographics and clinical characteristics.

	**N (%)**
**All patients**	44
**Gender**	
Men	23 (52)
Women	20 (46)
Nonbinary	1 (2)
**Race**	
White	21 (48)
African American or Black	15 (34)
Asian	3 (7)
American Indian or Alaskan Native	1 (2)
Unknown	4 (9)
**Ethnicity**	
Not Hispanic or Latino	23 (52)
Hispanic or Latino	21 (48)
**Religion**	
Christian or Catholic	26 (59)
Agnostic or None	9 (20)
Jewish	3 (7)
Islam	2 (4)
Hindu	1 (3)
Unknown	3 (7)
**Primary diagnosis**	
Sickle Cell Disease	14 (32)
Oncological Diagnoses	9 (20)
Congenital Defects Cardiac defects Non-cardiac defects	7 (16) 2 (4) 5 (11)
Genetic Syndromes	5 (11)
Cystic Fibrosis	3 (7)
Chronic Pancreatitis	3 (7)
Neuromuscular Diagnoses	2 (4)
Chronic Pain	1 (2)

### 3.2 Palliative care utilization

The median age that patients had their first adult palliative care encounter was 23.35 (IQR: 20.40–27.48) years. This encounter occurred significantly earlier if patients had previous pediatric palliative care exposure (19.63 versus 25.06, p = <0.001). The setting of the first adult palliative care encounter was an outpatient visit for 59 % (N = 26) of patients and an inpatient consult for 41 % (N = 18) of patients. The referral sources for adult palliative care outpatient visits were outpatient subspecialists (73 %, N = 19), primary care physicians (15 %, N = 4), pediatric palliative care providers (8 %, N = 2), and self-referral (4 %, N = 1). Patients often had multiple reasons for referral to adult palliative care services. The most common reasons were pain management (84 %, N = 37), symptom management (41 %, N = 18), psychosocial support (34 %, N = 15), and goals of care (14 %, N = 14) (Table 2). In our institution, a separate outpatient non-palliative pain management service exists and is focused on the management of chronic pain. Patients with pain previously treated by the palliative care team, such as in the inpatient setting, or with pain and other symptoms that are a result of serious/life-limiting illness are generally managed by palliative care rather than by the pain management service in the outpatient setting.

**Table 2:** Referral sources and reasons for adult versus pediatric palliative care.

	**Adult palliative care** N = 44	**Pediatric palliative care** N = 10
**Referral source** Inpatient *Inpatient team* *Emergency department* Outpatient *Outpatient subspecialist* *PCP* *Pediatric palliative care* *Self-referral*	18 (41 %) *18 (41 %)* *0 (0 %)* 26 (59 %) *19 (43 %)* *4 (9 %)* *2 (5 %)* *1 (2 %)*	100 (100 %) *9 (90 %)* *1 (10 %)* 0 (0 %) *0 (0 %)* *0 (0 %)* *0 (0 %)* *0 (0 %)*
**Referral reason(s)**^**†**^ Pain management Symptom management Psychosocial support Goals of care	37 (84 %) 18 (41 %) 15 (34 %) 6 (14 %)	5 (50 %) 5 (50 %) 4 (40 %) 4 (40 %)

^†^Referral reasons are non-mutually exclusive

All pediatric palliative care experiences were inpatient, because at our institution, pediatric palliative care exists only as an inpatient consult service. There is also a pediatric pain medicine team that provides inpatient consult services. Many patients had no prior pediatric palliative care or pediatric pain medicine encounters. In our study population, 23 % (N = 10) of patients had at least one prior pediatric palliative care consult, 36 % (N = 16) of patients had at least one prior pediatric pain team consult, 48 % (N = 21) had at least one prior consult from either the pediatric palliative care or pediatric pain team, and 11 % (N = 5) had both teams consulted in the past. Among patients with exposure to pediatric palliative care (N = 10), the average number of encounters (i.e., ED visits or hospital admissions) for which pediatric palliative care was consulted was 2.60 (SD: ± 1.84) per person. The initial consult sources were general inpatient pediatric teams (60 %, N = 6), pediatric intensive care units (30 %, N = 3), and the pediatric ED (10 %, N = 1). The most common reasons for referral to pediatric palliative care services were pain management (50 %, N = 5), symptom management (50 %, N = 5), psychosocial support (40 %, N = 4), and goals of care (40 %, N = 4) (Table 2).

**Fig. 2:**
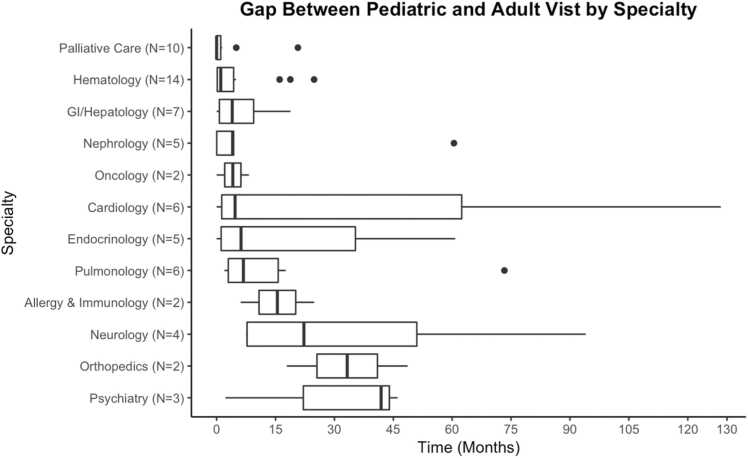
Gap between the last pediatric and first adult appointment by specialty, Gap in time (in months) between patients’ last pediatric and first adult subspecialty appointments, representing the amount of time they had a gap in care in that subspecialty**.** Note that the sample size (N) for each subspeciality is different, as each individual in the study saw different specialists based on their needs and diagnoses.

Regarding the transition from pediatric to adult services, the median gap in time between patients’ last pediatric and first adult palliative care encounters was 0 (IQR: 0–1.05) months. This amount of time, which represents a gap in care, is often used as a metric for success in transitioning from pediatric to adult specialties. The median transfer gap was lower for palliative care than it was for all other specialties, which ranged from 1.05 (IQR: 0.16–4.33) months (in hematology) to 41.88 (IQR: 22.08–43.99) months (in psychiatry). These transfer gaps were visualized for each specialty using box plots depicting the median amount of time between visits with interquartile ranges (Fig. 2).

### 3.3 Goals of care, code status, and advance directives

Patients’ GOC and code status conversations were documented. A GOC conversation was defined as a documented meeting during which a patient’s values, priorities, and overarching aims of medical care were discussed within the clinical context to guide decisions about the use or limitation of specific medical interventions.^13^ A code status conversation was defined as any meeting during which decisions about code status were explicitly discussed.

In our study population, 61 % (N = 27) of patients had a documented GOC conversation. The median age at which patients had a GOC conversation, estimated using time-to-event analysis, was 25.7 years old. Using a Kaplan-Meier estimator, the probability of having had a GOC conversation was 11.4 % by age 18, 31.8 % by age 21 %, and 53.8 % by age 26. A patient’s GOC conversation was likely to have occurred significantly earlier if pediatric palliative care was involved (18.9 years versus 25.7 years, p < 0.001). The most common setting for a first GOC conversation (N = 27) was an inpatient hospitalization (67 %, N = 18), followed by an outpatient specialist appointment (22 %, N = 6) or an outpatient primary care provider visit (7 %, N = 2). One patient had their first GOC conversation while in the ED (4 %, N = 1) (Table 3).

**Table 3:** Frequency of GOC, code status and HCP conversations and patient age at which they occurred.

	**All patients** (N = 44)	**Patients without pediatric palliative care services** (N = 34)	**Patients with pediatric palliative care services** (N = 10)
**Goals of care conversations**^**†**^ Average age Frequency	25.7 years 27 (61 %)	25.7 18 (53 %)	18.9 years 9 (90 %)
**Code status conversations**^**†**^ Average age Frequency	28.7 years 22 (50 %)	30.0 years 17 (50 %)	20.9 years 5 (50 %)
**Healthcare Proxy Identification**^**†**^ Average age Frequency	25.8 years 25 (57 %)	28.9 years 19 (56 %)	20.7 years 6 (60 %)

^†^Kaplan-Meier curves were used to estimate the time-to-event, reported in years of age, for GOC, code status and HCP conversations since they were outcomes of interest not completed by all participants during the study period. Comparisons were done via log rank test.

Fewer patients had an explicit code status discussion than had a GOC conversation. Only 50 % (N = 22) of the study population discussed their code status, and the median age of discussion based on time-to-event analysis was 28.7 years old. The probability of having had a code status conversation was 2.3 % at age 18, 18.9 % at age 21 %, and 43.2 % at age 26. Pediatric palliative care involvement was significantly associated with earlier code status discussions (20.9 years versus 30.0 years, p < 0.001). The setting of the first code status conversation (N = 22) was most often an inpatient hospitalization (82 %, N = 18), all other initial conversations occurred during outpatient specialist (14 %, N = 3) or ED visits (4 %, N = 1) (Table 3).

Any advance directives that were filed in a patient’s chart were recorded, including health care proxy (HCP) documents, do not resuscitate (DNR) orders, Medical Orders for Life-Sustaining Treatment (MOLST) forms, and living wills. Fifty-seven percent of patients had an appointed HCP (N = 25, median age estimated using time-to-event analysis 25.8), 18 % had DNR orders (N = 8, average age 24.74 (SD: ± 4.15) years, insufficient data for time-to-event analysis), 4 % had a MOLST form (N = 2, both age 30 years), and only 2 % had a living will (N = 1, age 17 years). Patients who had a pediatric palliative care consult were significantly more likely to file HCP documentation at a younger age (20.7 versus 28.9, p < 0.001) (Table 3).

### 3.4 Mortality

Thirteen patients (29 %) died during study period. The average age of death among these patients (N = 13) was 22.93 (SD: ± 3.25) years old. There were insufficient events to perform a time-to-event analysis with the entire study population. Four patients (31 %) had hospice designation, 2 were referred to hospice but died prior to enrollment (15 %), and 7 (54 %) did not have hospice referrals (54 %). Seven patients (54 %) died at home, 5 (38 %) died during an inpatient hospitalization, and 1 (8 %) died at an inpatient hospice facility. All patients who died had a documented goals of care conversation before death, and 92 % (N = 12) of patients had specifically discussed their code status preferences. Goals of care and code status conversations were more common in patients who died during the study period than those who lived (100 % versus 45 %; 92 % versus 32 %). The median time from initial GOC conversation to death was 3.6 (IQR: 1.56–51.90) months. Thirty-one percent of deceased patients had their first GOC conversation either during the hospitalization in which they died or, if they died outside the hospital, in the hospitalization immediately preceding their death.

### 3.5 Acute care encounters

We estimated the incidence of ED visits, hospital admissions, inpatient days, and total acute encounters (ED visits plus admissions) per year before and after patients’ 21st birthdays, as 21 years is the age when the pediatric to adult ED transition occurs at our institution. The median incidence of ED encounters per year (0.63 versus 0.93, p = 0.57) and hospitalizations per year (1.06 versus 1.71, p = 0.09) increased pre- to post-21st birthday, but this trend did not reach statistical significance. The median incidence of total acute care encounters (1.56 versus 3.38, p < 0.001) and inpatient days (8.84 versus 14.77, p = 0.01) both significantly increased after patients’ 21st birthdays.

### 3.6 Missed appointments and loss to follow up

The unadjusted incidence of missed appointments per year before and after each participant’s 21st birthday was calculated. The median number of missed appointments per year significantly increased after the age of 21 years (0.13 versus 1.54, p < 0.001). No patients in our study sample were lost to follow up, which we defined as having no in-network encounters for ≥ 180 days without documentation of patient death or transfer to an outside hospital.

## 4 Discussion

### 4.1 Benefits of early palliative care utilization

Our main outcome of interest was utilization rates of pediatric palliative care services in patients for whom these services would be appropriate. Most patients in our cohort were diagnosed with a primary condition that made them eligible for palliative care services perinatally, yet the involvement of palliative care services for these patients before adulthood was limited. Only 23 % of patients had a pediatric palliative care encounter, and the median age of exposure to adult palliative care was 23 years, representing a gap in services delivered during childhood, adolescent, and young adult years. This is consistent with a national trend of pediatric palliative care underutilization.^14^, ^15^, ^16^, ^17^

Our study supports the argument that earlier involvement of palliative care would be beneficial for young patients with complex, chronic illnesses, as it facilitates earlier discussions about patients’ treatment preferences. Overall, the documented incidence of goals of care and advance directive conversations was suboptimal in our patient population. Furthermore, these conversations were overdue among patients who died, with a median time of 3.6 months between the initial GOC conversation and the patient’s death. However, patients who engaged with the pediatric palliative care service had significantly earlier GOC conversations, code status discussions, and HCP identification.

It is known that when patients have advance directives, they are more likely to receive care that is concordant with their preferences.^18^ The standard of excellence in caring for pediatric patients with life-limiting illnesses is to have advance care planning decisions early, outside of a medical crisis, and before the patient is at imminent risk of dying.^19^ Research is limited in the AYA population, but in adult populations, evidence shows that most patients complete an advance directive more than a year prior to their deaths, and that late advance directives result in more aggressive care and incomplete, poorly communicated decisions.^20^ In our population, the majority of initial GOC and code status conversations occurred during inpatient hospitalizations, and almost one third of patients who died had their first GOC conversation during the hospitalization that either coincided with or immediately preceded their death. Expanding the settings where these conversations are occurring and improving their incidence and timing are areas for improvement.

### 4.2 Role of pediatric palliative care in healthcare transitions

We propose that pediatric palliative care involvement also helps patients with the transition to appropriate adult palliative care services. Patients who had a pediatric palliative care consult had their first adult palliative care encounter significantly earlier than patients who did not (19.63 versus 25.06, p = <0.001). Notably, the median transition gap between pediatric and adult palliative care services was 0 months, which was shorter than the gap for any other specialty service.

Adult palliative care services were initiated most frequently in the outpatient setting (59 %), originating as consults from outpatient subspecialists and primary care providers. This suggests that the lack of outpatient pediatric palliative care services at our institution may contribute to the gap in palliative care services for children and young adults. This is a broadly applicable finding, as most of the current focus within the specialty of pediatric palliative care is centered around inpatient models of care.^16^, ^21^, ^22^ Fortunately, there is survey data that suggests that outpatient pediatric palliative care services are expanding.^23^ There is growing research in general on how to improve timely referrals to outpatient palliative care, a topic that is beneficial for both adult and pediatric providers.^24^

Differences in the setting in which pediatric and adult services were provided (i.e., inpatient versus outpatient) may have affected the reasons for which patients were referred to palliative care. The reasons for referral were similar between the adult and pediatric services, but the frequencies were different. Adult palliative care, which included both inpatient and outpatient services, was consulted more frequently for pain management (84 % versus 50 %). Pediatric palliative care, which existed only as an inpatient service, was consulted more frequently for goals of care (40 % versus 14 %).

The changes in incidence of missed appointments, ED visits, hospital admissions, inpatient days, and total acute care encounters before and after patients’ 21st birthdays are metrics often used to assess the success of the transition to adult care. In our study population, the median incidence of acute care encounters, hospital days, and missed appointments significantly increased after patients reached the age of 21 years, suggesting a lack of success in the transition from pediatric to adult services. This indicates potential room for improvement in the transition process for chronically ill patients. Based on the data discussed above, we believe there is a role for palliative care services in these transitions. To our knowledge, there are few structured transition programs that actively involve palliative care services.

### 4.3 Study limitations

This study has several limitations. First, the sample size is small and located within a single hospital system, affecting power, generalizability, and subgroup analyses. Second, events that occurred at outside hospitals could not be included in analysis, and thus, may have confounded the results. Third, the limited scope of pediatric palliative services available within our hospital—where they exist only as an inpatient consult service—limits our ability to meaningfully compare the utilization of adult and pediatric services. Future research may benefit from a larger sample at an institution or institutions that are associated with outpatient pediatric palliative care services. Fourth, our study does not account for differences in patient life expectancy, and it is reasonable to believe that this variable may impact the healthcare transition process. Future research that incorporates life expectancy into the analysis may be useful. Additionally, we hope to further investigate patient and family perspectives on the transition through qualitative review of patient-provider conversations. A promising future quality improvement project would be the creation of a structured transition program that involves palliative care services.

## 5 Conclusion

The transition from pediatric to adult care is an especially vulnerable period for patients with complex illnesses. Our study adds to a limited body of knowledge about the adolescents and young adults who use palliative care services and are undergoing this transition. We demonstrate that patients struggle during the transition process, as evidenced by increased rates of missed appointments and acute care utilization as patients age into adulthood. We also demonstrate that pediatric palliative care services are underutilized for this population, and that pediatric palliative care involvement can have notable benefits, such as earlier discussions about patients’ values and preferences. These findings encourage further research.

## Funding statement

This research did not receive any specific grant from funding agencies in the public, commercial, or not-for-profit sectors.

## Ethical statement

Approval was granted by the Institutional Review Board of Columbia University Medical Center (7/16/2022, Protocol #AAAU2001).

## Declaration of Competing Interest

The authors declare that they have no known competing financial interests or personal relationships that could have appeared to influence the work reported in this paper.

## Data Availability

Data will be made available on request.
